# Embedding the Pillars of Quality in Health Information Technology Solutions Using “Integrated Patient Journey Mapping” (IPJM): Case Study

**DOI:** 10.2196/17416

**Published:** 2020-09-17

**Authors:** Stephen McCarthy, Paidi O'Raghallaigh, Simon Woodworth, Yoke Yin Lim, Louise C Kenny, Frédéric Adam

**Affiliations:** 1 Department of Business Information Systems Cork University Business School University College Cork Cork Ireland; 2 Cork University Maternity Hospital Cork Ireland; 3 Dept. of Women’s and Children’s Health Institute of Life Course & Medical Sciences University of Liverpool Liverpool United Kingdom; 4 INFANT SFI Centre University College Cork Cork Ireland

**Keywords:** health information technology, health care quality, data analytics, multidisciplinary research, mobile phone

## Abstract

**Background:**

Health information technology (HIT) and associated data analytics offer significant opportunities for tackling some of the more complex challenges currently facing the health care sector. However, to deliver robust health care service improvements, it is essential that HIT solutions be designed by parallelly considering the 3 core pillars of health care quality: clinical effectiveness, patient safety, and patient experience. This requires multidisciplinary teams to design interventions that both adhere to medical protocols and achieve the tripartite goals of effectiveness, safety, and experience.

**Objective:**

In this paper, we present a design tool called *Integrated Patient Journey Mapping* (IPJM) that was developed to assist multidisciplinary teams in designing effective HIT solutions to address the 3 core pillars of health care quality. IPJM is intended to support the analysis of requirements as well as to promote empathy and the emergence of shared commitment and understanding among multidisciplinary teams.

**Methods:**

A 6-month, in-depth case study was conducted to derive findings on the use of IPJM during *Learning to Evaluate Blood Pressure at Home* (LEANBH), a connected health project that developed an HIT solution for the perinatal health context. Data were collected from over 700 hours of participant observations and 10 semistructured interviews.

**Results:**

The findings indicate that IPJM offered a constructive tool for multidisciplinary teams to work together in designing an HIT solution, through mapping the physical and emotional journey of patients for both the current service and the proposed connected health service. This allowed team members to consider the goals, tasks, constraints, and actors involved in the delivery of this journey and to capture requirements for the digital touchpoints of the connected health service.

**Conclusions:**

Overall, IPJM facilitates the design and implementation of complex HITs that require multidisciplinary participation.

## Introduction

### Prior Work

Significant investment continues to be directed toward service reform strategies to deal with the sizable challenges facing health care sectors [1]. These challenges include, but are not limited to, an increasing demand for chronic care, shortages in skilled medical labor, and an aging population [2,3]. In the United Kingdom, the government pledged a £20.5 billion (US $27 billion) increase in the National Health Service’s budget between 2019 and 2024 to foster widespread performance improvements across both primary and secondary care with the aim of tackling these challenges [4]. This trend toward increased spending is likely to continue into the future as nations across the globe seek to deal with large-scale economic and demographic changes [1].

Health care service redesign through the adoption of health information technology (HIT) is being proposed as a means of increasing both the efficiency and effectiveness of health care services, reducing waiting times, and improving the standards of patient care [5,6]. In particular, connected health has emerged as a promising area of research for addressing some of the current challenges [7-9]. This blends the physical and digital realms by capturing real-time data from numerous connected HIT devices (eg, smartphone apps, weighing scales, blood pressure monitors, etc) to ensure that health care stakeholders (eg, patients, carers, clinicians, etc) are provided with timely, accurate, and pertinent information regarding the patient’s status [8,10]. Combined with advanced data analytics, connected health platforms can also contribute to the improvement of health outcomes through targeted and early interventions [11]. For instance, data analytics can provide clinicians with key insights derived from patterns in large patient data sets, which can in turn contribute to improved clinical decision making. This can help reduce decision makers’ reliance on *gut feeling* or intuition by fostering a data-driven, evidence-based approach to clinical decision making and decision support [12-14]. Connected health platforms, combined with the use of smartphone apps, also offer the possibility of deploying coaching on a broad scale to improve adherence and outcomes for those affected by a variety of conditions, such as diabetes [15-17].

However, Chen et al [18] noted that these targets can only be achieved through appropriately designed interventions. This requires inputs from all relevant stakeholders to design connected health solutions that not only fit the needs of patients [19] but also fit within the health care ecosystems and are viable and sustainable in the long term [18]. The mapping tool that we present in this paper is aimed specifically at eliciting and channeling the opinions and preferences of a varied group of stakeholders around the possible use of HIT across a medical pathway.

According to Doyle et al [20], there are 3 core pillars of health care quality, which health care reform strategies (including those involving connected health) must cater to, clinical effectiveness, patient safety, and patient experience. Their contention has been broadly supported by other researchers (for instance, the study by Anhang et al [21]), with their paper receiving over a thousand citations and many researchers adopting their 3-pillar framework. The core argument in this stream of research is that the relationship between patient experiences and other aspects of care is symbiotic and critical. We agree with the view that patient experiences are an integral aspect of care quality (even if they may not be directly related to clinical processes and outcomes [22]. We strongly agree that we need to understand how patient experiences are associated with the effective use of structures, the underlying health care processes, and the occurrence of health outcomes. This knowledge ought to be directed toward improving the efficiency and effectiveness of care [21]. Thus, in this study, we adopted the 3 pillars of health care quality by Doyle et al [20] as a guiding framework.

To date, health service reform initiatives have focused on measures of clinical effectiveness and patient safety, with patient experience receiving less attention [5,23]. It does not follow that an efficient and compliant service will mean a good patient experience. For instance, a patient might receive an appointment quickly, but their overall experience may be poor if, for example, they feel that their unique needs are not catered to. In most cases, connected health solutions involve patients who directly engage with apps, often in their homes or in the community. Given the absence of direct supervision, it is critical that the apps and devices are easy to use and that they promote appropriate, accurate, and safe usage. Generally, connected health solutions raise significant and new ethical concerns, which need careful consideration [24]. Therefore, it is crucial that their design considers all 3 central pillars of health care quality (clinical effectiveness, patient safety, and patient experience) in tandem [20,25]. Failure to consider these pillars may mean that key requirements and constraints are overlooked, leading to problems later—poor quality data, low utilization of health care services, ineffective decisions by health care professionals, or unethical use of data [20,26].

Although methods are available for exploring each pillar of health care quality in isolation, to the best of our knowledge, there is no single design tool currently in use that addresses all 3 pillars collectively, and more particularly in the context of technology-intensive and multidisciplinary fields such as connected health. This paper, goes some distance to address this shortfall by presenting a design tool we developed called *Integrated Patient Journey Mapping* (IPJM). This tool is primarily aimed at supporting the analysis and design of connected health apps. Inspired by the concept of journey mapping, it allows researchers and practitioners to simultaneously and explicitly consider the factors of clinical effectiveness, patient safety, and patient experience in tandem. The tool has primarily been validated through its use in a series of projects. In this paper, we focus on its use in a project called *Learning to Evaluate Blood Pressure at Home* (LEANBH) that involves the development of a connected health app focused on the investigation of preeclampsia, a disorder of pregnancy that can lead to a variety of adverse outcomes.

The remainder of the paper is structured as follows: On the basis of a review of existing literature, the Introduction section offers a background to the development of the mapping tool in the context of connected health and describes IPJM. The Methods section explains the methods, while the Results section provides results from the LEANBH project on the use of IPJM in a perinatal context. A discussion of the findings as they pertain to academic and practitioner communities is outlined in the Discussion section.

### Background

#### Connected Health and Data Analytics

Connected health has been defined as a novel, conceptual model for health care management “where devices, services, or interventions are designed around the patient’s needs, and health-related data is shared, in such a way that the patient can receive care in the most proactive and efficient manner possible” [10]. Connected health aims to provide all actors involved in the delivery of health care services with timely, accurate, and pertinent information around the patient’s current state of well-being [8,10,27,28]. This is made possible by the development of information technology (IT) platforms that seamlessly integrate numerous *connected* health devices, which allow real-time management and monitoring of patients’ well-being across different settings [28-30]. This has been made possible through the increasing availability of new wireless networks (eg, Wi-Fi, Bluetooth, and 4G or 5G networks) that enable high-speed seamless integration of connected health devices and secure data repositories for storing health-related data.

Connected health platforms also enable health care actors to take effective measures for managing the patient’s state of well-being by analyzing health data from these devices [10,30]. Collected data from connected devices can be continuously analyzed and shared to provide actors with key insights that allow them to take effective action. For instance, feedback can be derived from an analysis of a patient’s home-based blood pressure readings or blood glucose levels taken from wearable body sensors or connected devices that record patients’ vitals [31,32]. In addition, rule-based systems can be employed to act as *early warning systems* whereby health care professionals are notified when a patient’s vitals pass certain thresholds, as detailed in the relevant clinical guidelines [33].

Connected health solutions and data analytics support a proactive model of care in which all stakeholders are provided with critical feedback at key touchpoints between the patient, the connected health platform, and the health care service [10,34]. At the same time, this provides a clear opportunity to re-engineer relevant pathways to boost their effectiveness while also leveraging leading-edge technology to reduce the transaction cost or increase the throughput of key health care services. However, the mapping of these touchpoints can be a challenging task, given the complexity of the pathways as well as the ubiquity and diversity of patient data in connected health scenarios [35]. Existing modeling techniques often fail to identify the ideal placement and configurations of connected health solutions within the health care service network [35].

#### Central Pillars of Health Care Quality

Quality improvement is the primary goal of all modern health care service organizations, which strive for better patient health care outcomes, service performance, and professional development in the delivery of health care services [36]. According to Doyle et al [20], there are 3 central pillars that constitute health care quality

##### Clinical Effectiveness

Clinical effectiveness concerns the improvement of the current clinical practices and their related health care service outcomes [25]. Clinical effectiveness can be improved through the identification of nonvalue adding steps that fail to directly improve the quality of patient care [37]. Workflow analysis can help improve the effectiveness, efficiency, and efficacy of clinical services based on an in-depth understanding of the status quo [38,39]. For instance, workflow analysis can be undertaken to investigate and identify potential variations in service delivery and to identify issues such as bottlenecks and resource constraints.

##### Patient Safety

Patient safety aims to safeguard different dimensions of patient well-being through regulation and proactive measures in practice [25]. The health care sector is a highly regulated environment, which demands that patient safety is taken into consideration in service reform initiatives. Examples of the constraints that ought to be considered when addressing patient safety include medical protocols and clinical guidelines (eg, the National Institute for Health and Care Excellence guidelines), ethical standards (eg, the Hippocratic Oath), medical device certification (eg, Food Drug Administration approval in the United States and Conformité Européene (CE) Marking in the European Union), and data protection (eg, General Data Protection Regulation). These factors act as guide rails that aim to improve patient safety [40].

##### Patient Experience

Patient experience centers on a patient’s “personal interpretation of the service process and their interaction and involvement with it during their journey or flow through a series of touchpoints” [41]. Zomerdijk and Voss [42] state that experiences are constructed based on the interpretation of encounters and interactions designed by the service provider. Although providers cannot directly offer an experience, they can create the foundational basis on which stakeholders (eg, customers, patients, and employees) can derive their own experiences. Although operational service quality looks at whether a service is delivered to its predefined specification, patient experience is based on the patient’s feelings, judgments, and perceptions of the benefits derived from the service [41,43]. Patient experience is a key factor in ensuring compliance with recommendations as patients are much more likely to disregard or abandon tools and practices if they contribute to a poor experience. Patient experience must also be considered from an ethical viewpoint where patients must be fully aware “of the nature, scope, and granularity of data collected and what information they are actually consenting to provide” [24].

However, although some methods for improving clinical effectiveness and managing patient safety are relatively well established in the health care sector (eg, process mapping, service blueprinting, etc), methods for enhancing patient experience are less entrenched, particularly within connected health [5,23,35]. The following section looks at journey mapping as a patient-centric tool for designing health care service reform.

### Journey Mapping

Journey maps have been used in several areas to offer pictorial illustrations of complex processes or interactions that would otherwise be difficult to apprehend. Howard [44] noted that journey maps evolved from the field of service design when designers sought to re-engineer or optimize the service delivery of organizations or developed blueprints for new services (see the study by Stickdorn and Schneider [45]).

In particular, journey maps can be used to depict the health care service from the perspective of different actors, such as patients [37,42,46]. In the case of the patient, they are based on mapping consecutive *touchpoints* between the patient and the service, the nexus of where patient experience is actively shaped [23,42,47]. They see the relationship between the patient and service organization as something emergent, dynamic, and ubiquitous within the larger context and go beyond the more static view provided by other service design methods [42]. Percival and McGregor [48], for instance, proposed a mapping technique that includes a number of layers: staff roles, processes, information creation or movement, HIT solutions, IT infrastructure, patient needs or practice guidelines or policies, and metrics. Journey maps incorporate both physical and emotional aspects of the patient’s journey with the aim of capturing and shaping the patient’s behavior, feelings, motivations, and attitudes across the episodes of care, taking into account such important factors as the environment or context. They also help professionals to visually externalize their disciplinary knowledge and collect multidisciplinary insights. This promotes alignment but also empathy toward patient groups by placing the patient at the heart of the modeling process [49] and by creating a visually compelling story of the patient’s experience [43].

User representations are developed to categorize and personify different target groups through the description of fictional users, that is, name, picture, personal background, and goals. User personas involve creating representations of typical users to help design teams to better understand and take account of the mental models of these groups, that is, their expectations, prior experience, and anticipated behavior [50]. LeRouge et al [50] stated that user personas address the limitations of common modeling tools such as Unified Modeling Language diagrams by integrating the conceptual model of users, their cognitive structures, and present behavior that drives health care thinking, future behavior, and demand.

Journey maps can be combined with user personas in the requirements gathering process to direct increased attention toward patient experience. The added contribution of personas to journey maps is that instead of being static representations of demographic profiles, they offer dynamic views of customers and users’ experiences in their interactions with current and proposed products and services. The combined approach can then be employed to make design decisions and evaluate design solutions according to the unique needs of each persona. This stimulates creativity among team members when trying to address user needs and usability across numerous different real-life scenarios [51]. Critically, a small number of personas have been found to support the consideration of large, diverse populations, making the concept particularly useful for health care scenarios [52].

### Developing Complex Apps

The area of HIT development has received considerable attention over the last 40 years. This time has seen the emergence of increasingly sophisticated platforms and development environments. Recently, the availability of cloud-based solutions, smart interconnected devices, and mobile apps has unleashed the potential for connected health apps. Unfortunately, these benefits can often be offset by the complexity and cost of developing connected health apps. The set of required development skills is becoming increasingly specialized, as is the complexity of the project management of the multidisciplinary teams required when developing such solutions. Mapping tools might be a useful approach for building cohesion within such teams, but at the same time, they must be understandable by diverse groups and professions to ensure that shared knowledge can be nurtured during the development process.

In the following section, we describe IPJM, a visual tool developed to help design teams to meet these challenges and to understand how to best reconcile the sometimes divergent requirements arising out of the need for clinical effectiveness, patient safety, and patient experience when designing connected health solutions. IPJM is also intended to promote harmonious team performance by negotiating and finding the right balance between the somewhat competing needs of different groups. This requires collaboration between different competencies on multidisciplinary teams. It also requires the management of conflict, which is likely to emerge from a comprehensive consideration of all viewpoints [53-55]. As a result of using IPJM, we hope that robust and high-quality designs will emerge for the solutions being considered.

### IPJM

**Figure 1:**
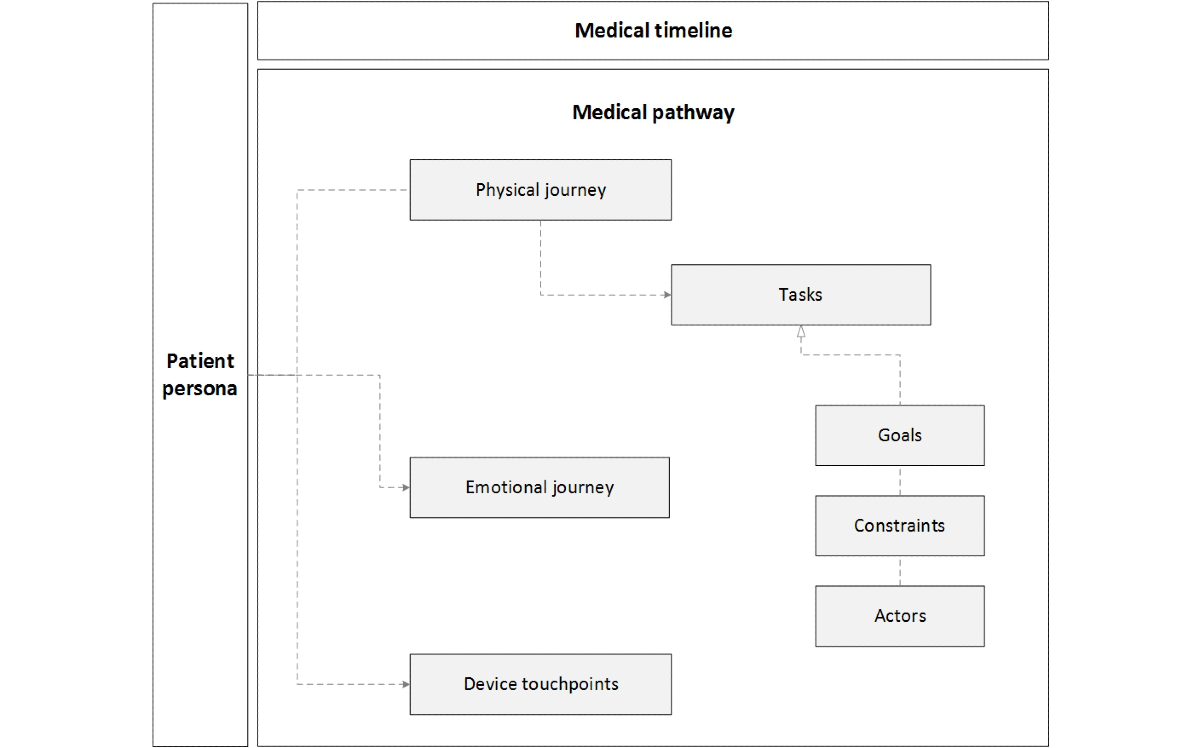
Integrated Patient Journey Map Ontology.

The IPJM tool was built using an ontology that conceptualizes the journey of a patient along a medical pathway. The ontology aims to promote a common vocabulary [56] among multidisciplinary design teams based on the 3 core pillars of health care quality. It captures the key elements of the journey: the structure of elements, relationships between elements, and implicit rules that govern the behavior of elements [57]. The ontology depicted in Figure 1 is provided in the literature. In addition, it has been validated through qualitative feedback from a number of projects that involve the use of IPJM, including the LEANBH project, which is described in the Methods section of this paper.

The ontology is split into 3 main areas: the *patient persona,* the *medical timeline*, and the *medical pathway*. First, the *patient persona* provides a characterization of a user group under consideration (eg, an expectant mother who is at risk of hypertension) and is inextricably linked to all other elements of the ontology. The *medical timeline* adds a temporal aspect to the episode of care by dividing it across a defined time frame (eg, the weeks of a pregnancy). The *medical pathway* centers on the consecutive events or steps in the episode of care [46] and consists of 7 subcomponents that are defined and described in Textbox 1. In particular, the *medical pathway* describes the *physical journey,* the *emotional journey,* and the *device touchpoints* associated with an episode of care. The *physical journey* is further divided into *tasks,* and these tasks are further subdivided into *goals, constraints*, and *actors.*

**Components of the medical pathway.**

- Physical journey: maps the movement of the patient across an episode of care as she moves from one touchpoint to another in different settings (eg, patient’s home, general practitioner clinic, or emergency room) where the health care service is delivered and the patient experience is derived
- Emotional journey: shows how the patient’s experience changes as she moves through the different touchpoints
- Device touchpoints: lists the technological solutions utilized by the different actors (eg, doctor, general practitioner, and patient) at each touchpoint
- Actors: lists the stakeholders involved in the delivery of the health care service (eg, hospital doctors, general practitioners, and nurses)
- Task: details the tasks undertaken by each actor in the health care service delivery (eg, measuring the patient’s blood pressure and registering appointments)
- Goals: comprises the desired outcomes that actors aim to deliver when carrying out tasks (eg, clinical, operational, and administrative goals)
- Constraints: outlines the constraints such as treatment guidelines based on medical protocols, governance, safety, and clinical guidelines

In this way, the ontology provides the foundational basis for IPJM by outlining the context in which the patient journeys transpire. Going back to the underpinnings of the concept of journey maps, the mapping tool (through the use of the ontology) visualizes the journey of a persona facing a scenario. This can sensitize designers and developers to the intricacies of individual personas and scenarios and minimize the risk of designing for *normative* situations that do not reflect the real situations of actual patients. Commercial firms and public sector agencies have used such ontologies very successfully in seeking to develop interaction mechanisms with their customers and with members of the public who need to access their services, such as in the case of disabled people who have special mobility and cognition needs [58].

IPJM can be used to show the *as is* and the *to be* comparison between the existing medical pathway and the intended modified pathway enhanced with technology, devices, apps, and other new components and interactions. This ensures the tool’s usefulness for negotiation and communication of the design of the proposed connected health solution, especially between clinical specialists and designers or developers of the solutions. In seeking to make a *business case* for new pathways, the map can be used to demonstrate to relevant health care authorities the potential impact of proposed changes.

#### IPJM Template

**Figure 2:**
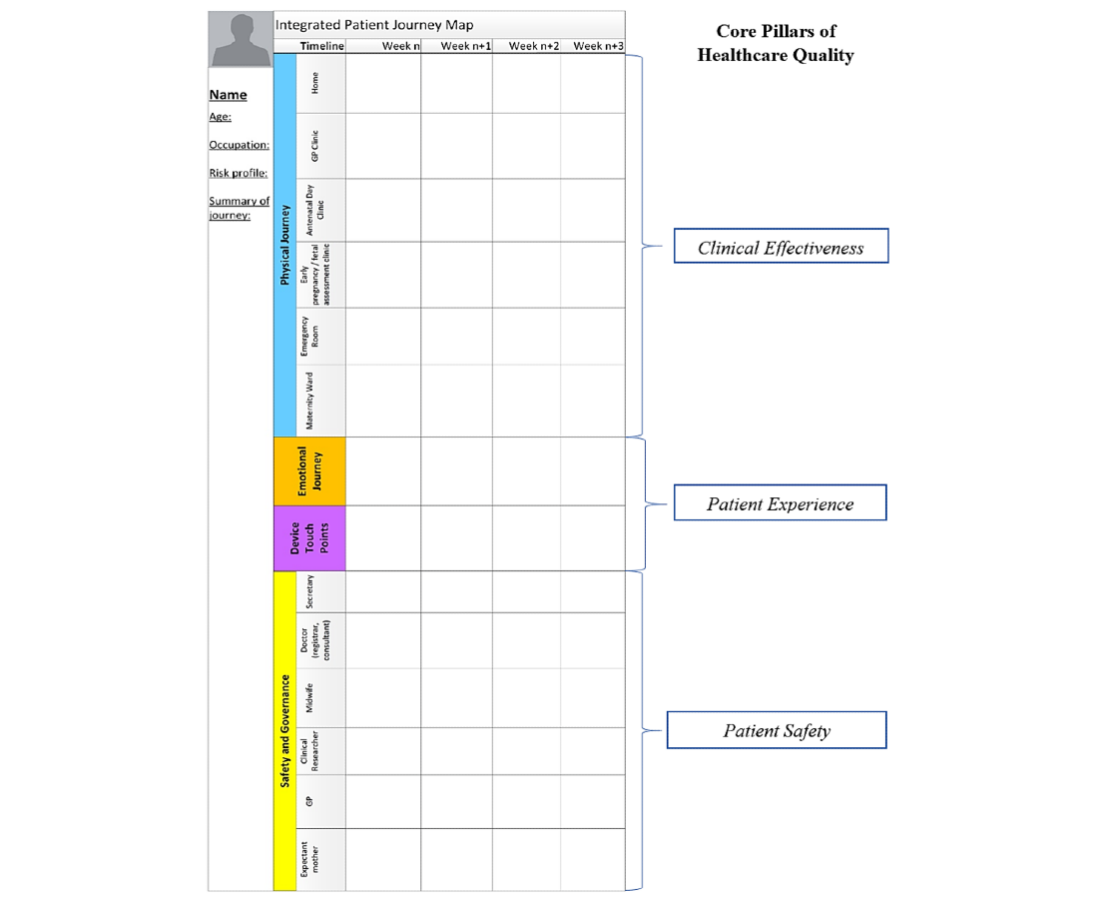
Base Integrated Patient Journey Map Template.

Building on this ontology, we iteratively designed and evaluated the visual elements of a journey mapping tool called IPJM. An example of a base template, constructed iteratively using the ontological components, is shown in Figure 2. The patient persona is situated on the left side of the template, the medical pathway and its subcomponents are positioned in the center, and the medical timeline is displayed horizontally on the top of the template. Tasks, goals, constraints, and actors are listed within the safety and governance component.

Each of these areas of the IPJM maps to the 3 core pillars of health care quality previously outlined in the Background section. For instance, the *physical journey* aims to provide insights into the clinical effectiveness of the health care service by plotting the sequence of steps involved in the delivery of care. This, in turn, can be used to examine the steps to identify those that do and do not add value to the health care service. The *emotional journey* deals with patient experience. This is based on the likely emotional response of the patient to individual steps in the health care service. Finally, *safety and governance* maps the aspects of patient safety based on the responsibilities of different actors and their associated regulatory constraints.

The device touchpoint area caters for the connected health context and maps the different *connected* devices and data analytic solutions that are employed by actors when delivering the service. For instance, one touchpoint between the patient and the health care service could involve the use of a smartphone app and a connected medical device for tracking and sharing data on the patient’s state of well-being. Another touchpoint could involve the use of data analytics by clinicians to gain insights into the patient’s state of well-being, forecasting potential health issues and intervening when required.

A design science approach was followed to ensure that there was a rigorous basis for the construction of the tool [59]. A description of the researchers’ approach to design science was previously presented in a study by McCarthy et al [60]. Following O'Raghallaigh et al [56], the design science approach consisted of 2 central activities: (1) *identifying and generating foundational abstract knowledge* from academic and practitioner literature to guide, explain, and justify the design approach and (2) *using and refining abstract foundational knowledge* in developing and evaluating prototypes through engagement with potential users of the tool. The approach thus sought to integrate both *design practices* (construction of the artifact supported by existing knowledge) with *design science* (generation of knowledge through the construction and evaluation of the artifact). For example, the initial version of the ontology was developed from a scientific understanding of the academic literature. On the other hand, the first version of the mapping tool was largely developed through practice.

Prototypes of the IPJM tool were evaluated using different techniques. Evaluation primarily focused on examining the use of the tool by design teams during projects focused on increasing health care quality (clinical effectiveness, patient safety, and patient experience). In addition, the general evaluation looked at IPJM as an analytical tool to support the collection of requirements for connected health apps. In the case of the LEANBH project, evaluation involved a multidisciplinary team of stakeholders working together to populate IPJM templates for 8 personas across diverse scenarios (such as white-coat hypertension, chronic hypertension, gestational hypertension, and preeclampsia). A separate template was used to map the journey for each persona facing a scenario. Post-it notes were used to *fill in* the components of the journey, and these were positioned across the 4 areas of the template. This approach allowed the journey to be easily modified by iteratively adding, moving, or removing the post-it notes. Different colored markers were used to connect and codify post-it notes and to indicate where changes needed to be made to the journeys based on discussion among the team members. Table 1 provides a summary of the evaluation techniques used during the LEANBH project.

The following section outlines the in-depth case study of the *LEANBH* project.

**Table 1:** Techniques used to evaluate the Integrated Patient Journey Mapping during the Learning to Evaluate Blood Pressure at Home project.

Data collection	Brief description	Purpose
Workgroup	Four full-day workshops involving a multidisciplinary group of stakeholders. The workshops focused on deriving requirements for a connected health system that would monitor the well-being of expectant mothers across different settings such as the antenatal clinic, general practitioner’s practice, and an expectant mother’s home	Exploratory design of the modeling tool
Semistructured interviews	Semistructured interviews each lasting about 1 hour were conducted with the 10 individual team members to gain further in-depth insights into the IPJM^a^ tool. Interviews were conducted with the principal investigator, project manager, 2 developers, a funded investigator, data architect, clinical lead, clinical researcher, research nurse, and the director of a commercial partner	Individual stakeholder’s subjective evaluation of IPJM
Analysis of supporting documents	A range of sources were used to ensure that IPJM considered clinical effectiveness, patient safety, and patient experience goals. This involved analyzing best practices around managing the patient pathway using sources such as the UK’s National Institute for Health and Care Excellence guidelines for managing hypertension during pregnancy. In addition, information requirements were investigated based on the Health Service Executive’s maternity health record in Ireland and Data Protection Act guidelines around health care research	Evaluation of the prototype’s ability to represent the current best practices

^a^IPJM: *Integrated Patient Journey Mapping*.

## Methods

### Case Study Approach

An in-depth case study approach [61] was undertaken to explore the use of visual tools for embedding health care quality in the design of connected health solutions. The in-depth case study in question followed the guidelines provided in studies by Yin [62,63]. It centered around the *LEANBH* project, a pilot research project that provides remote health care monitoring for expectant mothers to improve the detection and treatment of hypertension during pregnancy.

### The LEANBH Case Study

Hypertensive disorders in pregnancy (eg, preeclampsia and gestational hypertension) are a major cause of maternal and neonatal mortality and morbidity worldwide, accounting for 16% of maternal deaths in developed nations such as Ireland and 25.7% of maternal deaths in the developing nations of Latin America and the Caribbean [64]. In particular, preeclampsia is a hypertensive disorder of pregnancy characterized by high blood pressure (>140/90 mm Hg), the presence of protein in urine, and other associated symptoms such as headaches and edema, which can lead to serious complications during pregnancy [65].

The LEANBH project was a collaborative effort that involved organizations from academia, the health care sector, and the industry. The multidisciplinary project team consisted of a principal investigator, a project manager, a full-time and part-time developer, an analyst, and a data architect (which made up the *information systems* [*IS*] *subgroup*) and a clinical lead, a clinical researcher, and a research nurse (which made up the *clinical subgroup*). The primary goals of the project were to increase clinical effectiveness, patient safety, and patient experience in a perinatal care context. The project team was tasked with building a connected health platform that integrates several IT artifacts, including a smartphone app, a home blood pressure monitor, and a urine analyzer for use by expectant mothers. An electronic health record was included to capture vitals for use by clinicians. The project also aimed to develop novel forecasting algorithms for predicting the likelihood of gestational hypertension and preeclampsia.

The project was an observational study in which each patient followed the standard pathway and had access to both the standard care and the connected health platform. This simplified the ethical approval process, which was mostly concerned with providing complete and precise information to participants and in eliciting their consent on recruitment. This was achieved by creating a comprehensive patient information leaflet and assigning a dedicated research nurse to recruiting patients and training them in the use of the smartphone app, blood pressure monitor, and urine analyzer. Ethical approval was granted by both the University Clinical Research Ethical Committee and the Health Service Executive via the Hospital’s Local Information Governance Group Research and Audit Committee. The authorization covered 2 rounds of recruitment of 50 patients each: the first group was an initial low-risk group and the second group was a more representative group of pregnant women, including women with preeclampsia.

#### Data Gathering

Qualitative data were triangulated using 3 data gathering techniques: participant observations, interviews, and project documents. First, the lead author was granted exceptional access to the live project setting, which allowed him to carry out over 700 hours of in-depth participatory observations in the field for a period of 6 months (June 2015 to January 2016). Participant observations allowed the lead author to gain rich insights into peoples’ actions and directly observe events as they unfolded. In addition, semistructured interviews, each lasting about 1 hour, were then conducted with the 10 individual team members to gain further in-depth insights into the project. The interviews provided rich accounts of the subjects’ own words. Finally, the lead author also had access to project documents throughout the development phase, which included emails, reports, and project management outputs. These documents offered a concrete account of the phenomenon of interest.

#### Data Analysis

Content analysis [66] was used to organize data into common themes and triangulate findings from interviews, project documents, and participatory observations. The content analysis centered on both *reflection-in-action* and *reflection-on-action* [67], with clinicians and IT specialists asked to validate IPJM and the individual journey maps. This hybrid approach was in keeping with our use of the case study method, in an intrinsic rather than an instrumental mode [68].

The journey map was first evaluated through reflection-in-action, with participant observations by the lead author using vignettes. As noted by Denzin and Lincoln [69], “it is important to keep in mind that when conducting qualitative research, the researcher is the main tool for analysis.” Vignettes provided “a focused description of a series of events taken to be representative, typical, or emblematic in the case” [70]. Vignettes were used in the first instance as many parameters were emergent in our data analysis, and we wanted to stay as close to the data as we could. This technique allowed the researcher to produce, reflect, and learn from data around key moments in the *everyday life* of the project [70,71]. Gaining familiarity with the data, although arguably time consuming, was a positive aspect of the data analysis process and helped deliver a better artifact as well as a deeper understanding of its efficacy.

The efficacy of the journey map was also validated through reflection-on-action by analyzing interviews. To enhance the rigor in our data analysis, we used the computerized software provided by NVivo (QSR International) to analyze the interview transcripts. The lead author identified the codes of interest, including variables such as concepts and properties as well as the relationship between these variables [70]. As part of the data analysis and evaluation process, the researcher’s perception of variables and relationships, otherwise referred to as theoretical sensitivity, was influenced by a reading of literature. The lead author continuously reread interview transcripts and used NVivo to manage the coding inventory.

## Results

During the project initiation phase, the project manager organized 4-day-long participatory design workshops that aimed to build a collective vision for the project and to gather requirements for the connected health platform. These workshops involved stakeholders from the IS and clinician subgroups. During the workshops, the project manager encouraged the groups to work together in utilizing IPJM to map the physical and emotional journeys of pregnant women across the touchpoints of the proposed connected health service. In this way, IPJM provided a canvas for the groups to explore an improved antenatal pathway, technical considerations of the connected health platform, and the needs and capabilities of different stakeholders (eg, expectant mothers, clinicians, developers, nurses, midwives, and other health care practitioners). The groups used markers and post-it notes to dialogically work through potential challenges faced by personas in engaging with the proposed service. Owing to delays in the ethical approval process, the interdisciplinary team did not have direct contact with expectant mothers during this time.

**Figure 3:**
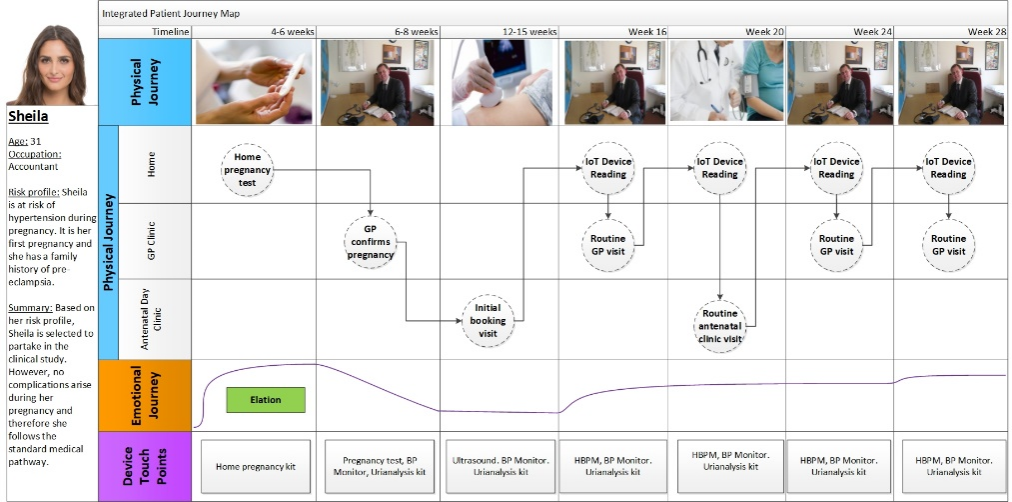
Snapshot of a Completed IPJM.

The project team used IPJM during successive workshops to superimpose the journeys of fictional personas of different expectant mothers who would use the connected health service. In total, 8 fictional personas were identified by the team to represent the different hypertensive disorders that can occur during pregnancy and the medical scenarios that can occur. This included *Sheila*, a 31-year-old first-time expectant mother at risk of hypertension during pregnancy because of a family history of preeclampsia (Figure 3). Her journey through the standard antenatal pathway was now complemented with her use of the proposed connected health solution. Other personas included *Denise*, a 25-year-old expectant mother who developed preeclampsia, and *Fiona*, a 29-year-old expectant mother who developed gestational hypertension.

The project manager viewed the use of fictional personas as vital in that they acted as surrogates for real expectant mothers in the participatory design phase. This gave a voice to individuals who could not be physically present in the room. As a result, IPJM helped to build a bridge between multiple voices both inside and outside the design process, including the missing voices of expectant mothers. Interestingly, these missing voices often acted as the *arbitrator* during group discussions. For example, when individuals disagreed on a point, they would often revert to asking one another what the personas would want. This challenged the siloed thinking of both the clinical and IS subgroups. Individuals would often speak out on behalf of one of the personas and assert how certain decisions would affect the physical and emotional journey of this expectant mother. One powerful example of this emerged during discussions around the journey of *Brenda*, an expectant mother who (due to the white-coat syndrome) is incorrectly diagnosed with gestational hypertension and admitted to the hospital. The group discussed the emotional impact that this event would have on Brenda and challenged itself to come up with ways in which the connected health platform could be designed to avoid the unnecessary hospitalization of Brenda.

IPJM proved useful in helping individuals to build a deeper understanding of the challenges faced by different users of the proposed connected health platform. An example is the case of an expectant mother, Denise, who had young children to care for during her pregnancy. Denise’s journey generated discussions around the challenges she would face if the smartphone app forced her to take blood pressure readings at strict time intervals, which could interfere with her childminding obligations. This challenged the group’s prior assumptions. They ended up altering the service to provide flexibility when blood pressure readings could be recorded.

IPJM enabled the group to develop a common language around the antenatal pathway. It became a powerful means of building a shared understanding. For example, the IS subgroup faced a steep learning curve to reach an understanding of the obstetrics domain and the various health care settings in which the connected health platform would be deployed. Similarly, clinicians had limited knowledge of the technical aspects of the connected health platform. IPJM challenged siloed knowledge around the clinical and technology pathways and helped bridge disciplinary boundaries. The synergies arising from this confluence of disciplinary knowledge were essential for highlighting IT and clinical challenges, both previously known and unknown. As pointed out by the developer:

It was useful. It was only when I walked through the journey map explaining how the [smartphone] app would work that I realised that others had different interpretations.

It also emerged that the IPJM tool was equally a means of generating shared commitment among the groups. Individuals later noted how participatory design activities using IPJM allowed the group to leverage the full range of capabilities possessed by the interdisciplinary group. As stated by the project manager, these activities represented a significant milestone where:

Technical concerns and clinician concerns were starting to be addressed as a unit as opposed to being two separate entities... For the first time people realised that the journey wasn’t a clinical journey, it wasn’t a medical journey, but neither was it a technological journey. It was all combined together.

In using IPJM, many individuals were largely unaware that they were generating requirements for the proposed platform. However, the analyst was able to capture requirements for the platform from the discussions taking place as individuals worked together in filling out the journey maps. The resultant journey maps became a record of all relevant design knowledge. Owing to the visual and instinctive nature of the journey maps, individuals were able to handle the complexity of the medical scenarios, whereas this would not have been possible if traditional modeling techniques had been used, as these require a level of familiarity that some individuals did not possess.

## Discussion

### Principal Findings

The findings suggest that IPJM can support multidisciplinary teams in exploring connected health solutions that consider the 3 pillars of health care quality: patient experience, clinical effectiveness, and patient safety [20]. It supports groups in understanding and negotiating conflicting requirements that can arise during transformational projects. This is achieved using journey mapping and user personas for graphically externalizing key domain knowledge. IPJM also promotes creative thinking around service reform goals and fosters dialogue among stakeholders, potentially leading to better solutions overall [72]. In addition, the ontology behind IPJM places constraints on groups, although it also allows the modeling to be easily adapted to different specialties, such as cardiology. The accessibility of the IPJM tool means that it can become a valuable boundary object [73,74], for discussions between multidisciplinary teams of stakeholders. For instance, IPJM enables ideas to be shared, interrogated, and visually externalized at both individual and group levels [56]. The use of mediums such as post-it notes means that the template is easy to use and modify as well.

Compared with other mapping tools, IPJM offers the possibility to focus on the comparison between the *as is* and *to be* versions of the pathway under study—this is a significant advantage in projects that pursue specific improvement targets. Its reliance on a visual grammar that does not require pre-existing knowledge (unlike other systems analysis and design approaches, such as Data Flow Diagrams or Value Stream Mapping, which require substantial training before participants can use them meaningfully) is also an advantage. The comparison with other techniques, such as Patient Journey Model architecture (PaJMa), the method proposed by Percival and McGregor [48], for instance, shows that IPJM manages to accumulate and represent a similarly broad variety of knowledge but with greater economy and without passing on the complexity of tasks and process steps onto the participants in the design process or, generally, onto the readers of the documentation. Both PaJMa and IPJM offer improvements over other mapping tools by allowing analysts to consider a much broader range of knowledge, but the use of personas in IPJM delivers a sharper focus on human aspects, such as the human experience, of patients, which is fundamental for connected health solutions that entail a context of use where patients are alone when using apps. In contrast to PaJMa, IPJM is likely to be more user friendly and more flexible in the case of first-time digitalization of medical pathways that involve mobile components that either patients or clinicians will use remotely.

IPJM can be used as a cornerstone for modeling health care service reform where stakeholders collaborate to derive an understanding of and commitment to requirements [75,76]. Textbox 2 summarizes the benefits inherent in the use of IPJM identified in its use during the LEANBH project.

**Strengths of *Integrated Patient Journey Mapping*.**

- Embeds pillars of quality: considers clinical effectiveness, patient safety, and patient experience in tandem
- Externalizes knowledge: allows stakeholders to externalize their domain knowledge and build a shared understanding
- Stimulates creativity: facilitates dialog between different stakeholders around developing creative solutions
- Accessible: easy for multidisciplinary stakeholders to understand, use, and modify
- Adaptable: can be adapted to the requirements of different contexts and specialties
- Emancipatory: facilitates the alteration of medical pathways and the development of solutions for addressing their shortcomings
- Educational: acts as a platform for communicating proposed changes, their impacts, and the intentions and ambitions of the teams

Beyond the benefits identified in Textbox 2, we argue that IPJM can boost team cohesion during the execution of novel design projects. Existing literature suggests that team cohesion is essential to the performance of teams consisting of individuals from diverse organizational and geographical backgrounds [77]. Team cohesion can be defined as the extent to which team members are aligned in their shared understanding of and shared commitment to project tasks, for example, the actions that individuals and groups seek to perform based on agreed plans [78,79]. Shared understanding involves a social process whereby the divergent knowledge of individuals is transformed to generate collaborative knowledge building [75,80]. Shared understanding is required to explore design spaces and overcome siloed thinking through the combination of existing knowledge in new ways. Meanwhile, shared commitment goes beyond shared understanding alone and requires team members to commit time, effort, and resources in line with proposals that have gained shared understanding [76,81].

Shared understanding and shared commitment are crucial to the success of projects involving stakeholders from different organizational and disciplinary backgrounds [54]. In the absence of both shared understanding and shared commitment, the perspectives and intentions of team members can become increasingly fragmented, as individuals may not even be aware of the intricacies of the issues around which they disagree [76]. IPJM provides team members with the opportunity to challenge assumptions embedded in *prebaked* project proposals and contribute diverse knowledge around the design of IT solutions. This helps ensure that design efforts promote both a shared understanding of users’ diverse needs and capabilities and a shared commitment to the delivery of solutions that cater to these needs. However, during the LEANBH project, not all group members were equally committed to leveraging the tools and to journey maps for modeling the problem domain and gathering requirements. This is a key concern as there is a possibility of a link between the involvement of stakeholders during the modeling process and their understanding of and engagement with the project overall. Therefore, future versions of the modeling tool need to consider how best to engage practitioners from different backgrounds so that the entire team rally around the journey maps and their validation.

### Conclusions

The health care sector is currently facing the monumental challenge of minimizing the costs associated with health care delivery while simultaneously improving quality. Connected health solutions can play a significant role in meeting this challenge by transferring health care delivery to the least expensive setting (ie, a patient’s home) in a way that does not compromise quality. However, the successful design of connected health solutions is far from a straightforward task, and the success hinges on a quality-centric approach being embodied during every step of the development lifecycle. At this point in time, health care systems around the world are seriously affected by their reliance on a one-to-one mode of care delivery, where patients often wait for weeks and months to see overstretched specialists. Crucially, connected health apps can allow clinicians to better care for more patients by giving them more frequent attention in a remote fashion and without the need for face-to-face visits far more effectively [8].

It is here that the use of design tools such as IPJM can offer significant value. This paper contributes theoretical and practical insights into how visualization tools can be used to embed the pillars of health care quality in the design of connected health solutions. For instance, case study findings suggest that IPJM can provide multidisciplinary teams with a canvas for designing connected health solutions tripartite goals of clinical effectiveness, patient safety, and patient experience. In particular, IPJM can help ensure that patient experience is given ample consideration when designing health care services, in tandem with more traditional concerns such as resource efficiency, waiting times, financial costs, and treatment efficacy. In particular, IPJM can help bridge the gap, which is often identified too late between the intended use of apps and the observed system-in-use postimplementation. Such gaps often lead to the occurrence of *silent errors* and require the complete rethinking of apps and devices at considerable expense in time and money, both of which are in short supply in the health care sector [82].

### Limitations and Future Research

However, IPJM is not without some limitations. For instance, IPJM does not make explicit reference to key performance indicators, such as throughput and waiting times, or other metrics, such as productivity and cost-efficiency, although these may be essential elements of the performance and success of the services being designed. This clearly applies to the scenario of a connected health solution being implemented to increase the throughput of a medical pathway, to deliver cost savings, and to improve visibility on patients’ conditions. Although incorporating this element in the tool would be useful, there is also a risk that increasing the level of detail may compromise the overall accessibility and reliability of the maps. As a result, it may be difficult to capture some of the inherent complexity in health care systems, that is, when a patient is transferred from a hospital during treatment. On the other hand, the tool can be adapted according to the unique context in which it is to be used to address any key elements that are missing. Its use within the context of specific pathologies and medical specialties has the potential to rapidly bring medical teams up a steep learning curve toward developing connected health care apps.

Specifically, in the case of our research, we encountered other limitations, although it may be unclear whether these were circumstantial or if they were likely to also occur in other cases and settings. We found it difficult at times to secure participation from certain groupings in some meetings. For example, clinicians sometimes found it difficult to commit time to use IPJM, as they felt they were too busy and that the journey maps were for the development team rather than for themselves. Resolving these misconceptions is essential to producing maps that are accurate and robust in the face of real-life scenarios.

Future research may also seek to develop a more interactive version of IPJM to provide a more accessible view of the patient’s journey. IPJM currently requires a large physical display to ensure that all components are visible and legible. During the project, we experimented with different display dimensions and orientations before deciding on an A2 portrait format. However, it may be necessary to consider whether certain elements need to be reorganized so that the tool can be displayed more easily across a variety of media and spatial dimensions. A software program that would allow users to drill down into subpathways and map components more effectively could also be a useful extension.

Clearly, there are cognitive and presentational limitations that apply to the mapping of macroservices, for instance, a national or even transnational architecture for managing a certain pathology or group of patients with dedicated needs. Although the mapping of such a broad pathway might be desirable or even essential as a communication tool for reaching a common agreement, evidently difficulties will arise when attempting to compile such a map where the need to be holistic and comprehensive might be traded off against the necessity for visual representations to remain comprehensible by most people and therefore useful. Setting some boundaries that accommodate both the need to capture the whole system as well as some of its key components will be useful, although our research does not provide clear avenues pertaining to how this may be achieved. Weick [83] characterized the *Bonini paradox* (by reference to Charles Bonini and his work on simulation, published in 1963 [84]) as illustrative of situations where models were proposed that were so complex in and of themselves that it was no easier to understand them than it was to understand the real world as observation could reveal it. We can hypothesize that the *Bonini paradox* applies to journey maps and that *die hard* attempts to capture a world without any ontological boundaries would only yield theoretically excellent but practically useless representations that would hamper design efforts rather than help. The need for ontological boundaries, such as those provided by the IPJM tool, is much needed and is underresearched. Future research on this topic should explore this new dimension.

## Abbreviations

HIT: health information technology
IS: information systems
IT: information technology
IPJM: *Integrated Patient Journey Mapping*
LEANBH: *Learning to Evaluate Blood Pressure at Home*
PaJMa: Patient Journey Model architecture
